# Decision-Making Dysfunctions of Counterfactuals in Depression: Who Might I have Been?

**DOI:** 10.3389/fpsyt.2013.00143

**Published:** 2013-11-08

**Authors:** Jonathon R. Howlett, Martin P. Paulus

**Affiliations:** ^1^Laboratory of Biological Dynamics and Theoretical Medicine, University of California San Diego, La Jolla, CA, USA; ^2^Department of Psychiatry, University of California San Diego, La Jolla, CA, USA; ^3^Veterans Affairs San Diego Healthcare System, San Diego, CA, USA

**Keywords:** major depressive disorder, decision-making, emotions, neurosciences, self concept, neostriatum, cognition, affect

## Abstract

Cognitive neuroscience enables us now to decompose major depressive disorder into dysfunctional component processes and relate these processes to specific neural substrates. This approach can be used to illuminate the biological basis of altered psychological processes in depression, including abnormal decision-making. One important decision-related process is counterfactual thinking, or the comparison of reality to hypothetical alternatives. Evidence suggests that individuals with depression experience exaggerated emotional responses due to focusing on counterfactual decision outcomes in general and regret, i.e., the emotion associated with focus on an alternative superior outcome, in particular. Regret is linked to self-esteem in that it involves the evaluation of an individual’s own decisions. Alterations of self-esteem, in turn, are a hallmark of depression. The literature on the behavioral and neural processes underlying counterfactual thinking, self-esteem, and depression is selectively reviewed. A model is proposed in which unstable self-representation in depression is more strongly perturbed when a different choice would have produced a better outcome, leading to increased feelings of regret. This approach may help unify diverse aspects of depression, can generate testable predictions, and may suggest new treatment avenues targeting distorted counterfactual cognitions, attentional biases toward superior counterfactual outcomes, or increased affective response to regretted outcomes.

## Introduction

Major depressive disorder (MDD) has a substantial negative impact on both individuals and society, with a lifetime prevalence of 16%, and a 12-month prevalence of 6% (1, 2), yet our mechanistic understanding of the behavioral and brain processes underlying this disorder is still incomplete. One approach for understanding depression draws on models of cognitive and neural processes derived from cognitive psychology and cognitive neuroscience. In this approach, depression is viewed as resulting from dysfunction of component processes. For example, it has been argued that depression involves alterations in memory processes (3, 4), attention (5, 6), mental schemas (7, 8), and the reward systems of the brain (9, 10). Decision-making is another process which may be altered in depression. Research in decision-making has brought together researchers from a number of disciplines including economists, psychologists, and neuroscientists. This review focuses on one specific process involved in decision-making: counterfactual thinking, i.e., the comparison of reality with hypothetical alternatives or “what might have been.” A wealth of literature has indicated that people compare the outcomes of their decisions with alternative outcomes which did not occur, and that these comparisons influence the value they place on the actual outcome (11, 12). Counterfactual comparisons can influence the decisions that individuals make by affecting the valuation of outcomes but also via the emotional reactions to the outcomes of their decisions. A special class of counterfactual thinking is *self-related*, involving counterfactual scenarios in which the individual made a different choice. One possible consequence of this type of counterfactual thinking is regret, an emotion which has been shown to affect decision-making (13). Studies have found that proneness to regret is correlated with depressive symptoms (14).

Depression has long been linked to abnormalities in self-representation. In particular, it is known that fragile self-esteem (self-esteem which is particularly responsive to external events) is an important risk factor for developing depression (15). This review will argue that fragile self-esteem plays a role in altered processing of self-related counterfactuals in depression. Specifically, the brittle self-representation in depression is more strongly perturbed when a different choice would have produced a better outcome, leading to increased feelings of regret. This in turn will cause a devaluation of the actually experienced outcome.

Understanding the connections between counterfactual thinking, self-esteem, and depression may yield valuable insights into the nature of depression. Specifically, it may be useful in attempts to construct a unifying framework for diverse aspects of depression, including developmental, social, environmental, cognitive, affective, and biological. Self-esteem, along with the broader self concept, may be particularly helpful as a unifying construct because it is intimately related to cognition, affect, and social relationships and is thought to play a mediating role between environmental stressors and the affective and biological responses to those stressors (16). The self has been central to discussions of depression within diverse theoretical orientations. Investigating how self-esteem affects decision-making in depression may further help to elucidate the role of self concept in cognition, affect, and behavior in this disorder.

This review synthesizes an array of research from social and personality psychology, cognitive psychology, and neuroscience. The aim is to yield new insights into the role of counterfactual thinking in depression, a topic that has received relatively little attention. The model of altered counterfactual processing in depression proposed here could be used to generate new, testable predictions about counterfactual thinking and regret in depression.

The review involved searches for publications on decision-making, counterfactual thinking, self-esteem, and depression. Given the scope of each topic, a comprehensive review of all topics was not feasible, so a selective review was performed with emphasis on research relating the areas to one another and to neuroscientific findings. First, the role of counterfactual thinking in decision-making will be examined. Second, an overview will be provided of research on self-esteem. Third, these constructs will be linked to dysfunctions of decision-making in depression. Fourth, a simple model of the interaction between self-esteem and counterfactual thinking in depression will be proposed.

## Counterfactual Thinking

### Conceptualizing decision-making

In order to understand the role of counterfactual thinking in decision-making, it is helpful to review the process by which decisions are made. One approach to conceptualize decision-making is to distinguish between the different phases of the choice process. Prior to a decision, the decision maker is presented with various options or choices. Accounts of human decision-making typically argue that, during this phase, the decision maker will anticipate various possible outcomes of each choice (17–19). After this phase, a choice is selected. Many accounts of decision-making assume that choice selection is based, in part, on the subjective *value* of the various outcomes (20, 21). After making a decision, the decision maker often learns the outcome of the decision, and may additionally learn or imagine the alternative outcomes of the options which were not selected. The decision maker may then have an emotional response to this information. Importantly, some accounts of decision-making emphasize the idea that, prior to selecting a choice, decision makers will anticipate not only the possible outcomes of the choice, but the *emotional responses* to the various outcomes. This *anticipatory affect* then helps to guide decision-making (22, 23). Various factors can contribute to the emotional response to an outcome other than the outcome itself. For example, the emotional response may be affected by alternative possible outcomes which did not occur (11, 22), by the individual’s expectations (22), or by whether the outcome results from an action vs. an inaction (24).

### Overview of counterfactual thinking

Evaluating the actual outcomes of past actions and the anticipated outcomes of future actions is central to the process of decision-making. Studies of human decision-making have shown that individuals evaluate outcomes in comparison to other possible outcomes, rather than evaluating the outcomes in and of themselves. When experiencing the result of a particular choice, an individual can examine how this outcome compares to the imagined outcomes of other possible choices. For example, if a choice has led to a small gain but there was an opportunity for a significantly larger gain, individuals may process the small gain as a loss compared to the missed superior outcome. This evaluation of hypothetical alternative outcomes in relation to the experienced and actualized outcome is referred to as counterfactual thinking. Counterfactual thinking is ubiquitous in humans and is often framed in phrases such as “what might have been,” and “if only.”

Research has identified some of the factors that determine whether an individual will engage in counterfactual thinking at a particular time, and what types of counterfactual outcomes are considered. These investigations have suggested that counterfactual thinking is influenced by whether an outcome is expected or unexpected as well as by the focus of an individual’s attention. For example, Kahneman and Tversky have found that “close calls,” such as barely missing a flight, often lead people to consider counterfactual outcomes (e.g., the possibility of having made the flight). The same authors found that people often imagine counterfactual scenarios which remove a surprising element from what actually occurred (25). For example, one person might be delayed by traffic on a rural road, while another is delayed by traffic in a busy city at rush hour. The person on the rural road would be more likely to focus on a counterfactual situation in which there was no traffic because the experience of traffic in this context is surprising and unexpected. Counterfactuals can also be constructed by manipulating the events upon which people are focused (25). If someone is focused on their own behavior, they might generate a counterfactual in which they acted differently – a *self-related* counterfactual. In the same situation, someone focused on the behavior of others might generate a counterfactual in which another person acted differently. In summary, people typically engage in counterfactual thinking after a surprising event; they compare the actual outcome to an alternative that would have been less surprising. Additionally, people who are focused on themselves and their own behavior are more likely to imagine themselves having behaved differently, a process known as self-related counterfactual thinking.

### The affective experience

The consideration of counterfactual outcomes can influence a person’s experience of the outcome and generate specific emotional responses; among these are disappointment, regret, and relief. In disappointment, an outcome is compared unfavorably to an alternate possible outcome. Disappointment is contrasted with relief, which occurs when an outcome is compared favorably to an alternate possible outcome. One study examined disappointing wins (when a person wins money, but could have won more) as well as relieving losses (a person loses money, but could have lost more). By using self-report measures of emotions, the study found that both disappointing wins and relieving losses result in mixed feelings – both positive and negative emotions co-existing at the same time (12). In summary, both disappointment and relief involve comparison of an actual outcome to a superior or inferior one that did not occur.

Regret, an emotion that is frequent when an individual engages in counterfactual thinking, occurs after making a choice and later learning or imagining that a different choice would have produced a better outcome. Like disappointment, regret involves comparison of reality to a superior alternative outcome, however, regret involves counterfactual situations in which the individual acted differently. Regret therefore involves self-related counterfactual thinking (26). Studies have found that regret is specifically associated with a sense of responsibility, whereas disappointment occurs in the case of a bad outcome for which a person does not feel responsible (27, 28). People are also more likely to anticipate regret when they expect feedback about their actions; if a person will never know what would have happened if they made a different choice, they will be less likely to feel regret. Studies have shown that the expectation of feedback has a substantial effect on decisions by increasing avoidance of anticipated regret (29, 30). For example, if people expect to learn the outcome of a lottery whether they play or not, they will be more likely to play the lottery; this is thought to be due to the fear of regretting the outcome if they choose not to play and later learn they would have won. Regret is a highly aversive emotion; a study using self-report measures of satisfaction with outcome in a simple gambling task found that regret induced a more negative rating than disappointment for an equivalent monetary outcome (31). Overall, the research on regret has indicated that it is an aversive emotion in response to discovering that a different choice would have led to a better outcome.

### Dysfunctional patterns

Altered counterfactual thinking has been linked to a number of dysfunctional patterns of cognition and behavior. One such pattern is maladaptive perfectionism, a desire for a “perfect” outcome that results in significant negative affect when such an outcome is not achieved. Maladaptive perfectionism is associated with the propensity to focus more heavily on hypothetical alternative outcomes which were superior to the actual outcome (32). Maladaptive perfectionism has been associated with a wide range of mental illnesses including depression (33–37), anxiety disorders (38), obsessive compulsive disorder (39), and eating disorders (40–42).

Counterfactual thinking has also been linked to rumination, a negative and repetitive thought process. It has been suggested that some individuals experience ruminations about what might have gone better, resulting in negative affect (43). Some individuals may experience a “vicious circle” in which counterfactual thinking creates negative affect, which then leads to more counterfactual thinking (44). One recent study showed that individuals with more negative affect tended to spontaneously generate more counterfactual thoughts in a fictional diary entry (45). Rumination is closely linked with depression (46).

In summary, comparison to counterfactual outcomes has an important effect on how real outcomes are valued. When things could have gone better, people feel negative emotions such as disappointment or regret. Regret is distinct from disappointment in that it involves *self-focused* counterfactuals, i.e., those in which the individual acted differently.

## Neural Substrates Underlying Counterfactual Thinking

### Reward, decision-making, and the brain

The following section will provide evidence that counterfactual outcomes modulate responses in reward-sensitive brain areas. Before reviewing this evidence, it will be useful to very briefly review the construct of reward as it relates to neuroscience and decision-making. It must be emphasized that a comprehensive overview of this topic is outside the scope of the current review. Reward is a complex process, involving anticipation as well as consummation of a rewarding stimulus, which may recruit distinct brain regions (47). The term reward also encompasses both a subjectively pleasurable state as well as a reinforcing effect on behavior (48). A body of evidence now exists showing rewards from a diverse array of separate domains (e.g., financial rewards, social rewards, food and drink, sex, entertainment, and drugs of abuse) appear to be processed by the same brain networks, including the striatum and the medial prefrontal cortex (mPFC), including the orbitofrontal cortex (OFC) and ventromedial prefrontal cortex (vmPFC) (20, 21). It has been argued that these brain regions encode a common, domain-general representation of “value” which allows people to rank their preferences and choose between diverse outcomes (such as foregoing food in favor of an addictive drug) (20, 21). For this reason, reward and decision-making are closely linked. Specific subregions of reward-related brain areas may have specialized functions. For example, evidence has indicated that lateral OFC is activated after a punishing outcome, while medial OFC is activated after a rewarding outcome (49). Lateral OFC is also more likely to be activated when an action is made which requires suppression of previously rewarded behaviors (50) and when unsteady associations of stimuli and outcome require response shifts (51). An alternative account suggests that the medial OFC tracks intrinsic or short-term value, while the lateral OFC tracks extrinsic or long-term reward value (52). The medial prefrontal cortex (PFC) has also been shown to exhibit functional sub-specialization, with the more ventral regions showing more of a role in reward-related tasks, while the dorsal regions are involved in perspective-taking and episodic memory retrieval (53). Finally, the anterior cingulate cortex (ACC) also appears to have regional specialization, with the rostral portion involved in emotional processes and the dorsal portion involved in cognition (54, 55).

In addition to a set of brain areas, research into the neural basis of reward and decision-making has also implicated a set of neurotransmitters in these processes. While a complete overview of this literature is outside the scope of this review, a brief discussion of the most significant findings is warranted. Of the neurotransmitter systems that may be involved in decision-making, the most important is likely dopamine and its role in reward processes. A number of hypotheses have been put forward relating dopamine to reward processing, including the idea that dopamine contributes to the hedonic aspects of rewarding stimuli, that dopamine codes for a “reward prediction error” signal that guides learning, and that dopamine signals the incentive salience of a stimulus, thereby motivating the pursuit of rewards (56). While a full discussion of these hypotheses is outside the scope of this review, it is helpful to note that dopamine appears to play a key role in reward processes. Furthermore, dopaminergic neurons in the midbrain are known to project heavily to the striatum (57) and to the PFC and ACC (58). The striatum in turn is connected to frontal areas including the OFC, ACC, and mPFC in a series of circuits (59). Dopamine has been linked to value-based choice (60) and risk-based decisions (61). Besides dopamine, another neurotransmitter which may be important in decision-making is norepinephrine, which appears to signal uncertainty with respect to decision outcomes (62). Other neuromodulatory neurotransmitters, such as serotonin, also likely play a role, although the function is somewhat less clear at this time. For example, serotonin may be involved in consideration of delayed rewards (62).

Despite some limitations of fMRI, including limited temporal resolution (63), susceptibility artifacts (64), and physiological and non-physiological noise limiting its signal to noise ratio (63), significant progress has been made to identify the role of certain neural systems in counterfactual decision-making, which will be reviewed in the next section.

### Counterfactual effects on reward-sensitive brain areas

Functional neuroimaging and lesion studies have begun to elucidate the brain basis of counterfactual thinking and its role in decision-making. Counterfactual thinking appears to depend on the OFC (31, 65–67). Several other areas, including the sublenticular extended amygdala and striatum show levels of activation which depend on both the actual outcome and the alternative, counterfactual outcome (68). One imaging study used a “wheel of fortune” gambling task in which different outcomes (gains and losses) were available on each trial. The study found that the activity in the nucleus accumbens and sublenticular extended amygdala in response to winning $0 depended on what the counterfactual outcome would have been: when the non-obtained outcome would have been a loss, the nucleus accumbens and sublenticular extended amygdala showed more activation in response to a $0 outcome than if the non-obtained outcome would have been a gain (68). This suggests that these brain structures respond not merely to the monetary outcome, but to the outcome in contrast to the counterfactual outcome. A $0 outcome is treated more like a gain when contrasted with a counterfactual loss, but is treated more like a loss when contrasted with a counterfactual gain. Similarly, another study found that several reward-sensitive brain areas including the striatum respond to a monetary reward relative to the alternative possible outcomes. This study used a simple gambling task in which subjects selected one of three cards to obtain a monetary outcome. Trials were divided into gain trials, in which the best outcome was a monetary gain and the worst outcome was no gain, and loss trials, in which the best outcome was no loss and the worst outcome was a loss. A large win in a gain trial (when the alternatives were a smaller gain or no gain) resulted in similar levels of activation as did no loss in a loss trial (when the alternatives were a large loss and a smaller loss) (69). In summary, activity in reward areas depends not only on the actual outcome but the alternative outcome as well. When the alternative outcome would have been better, activity in reward areas is decreased; when the alternative would have been worse, reward areas are more active. These neuroimaging findings parallel the behavioral and self-report studies reviewed above showing that the subjective value of an outcome depends on the unrealized alternative outcomes.

### The neural basis of regret

Several studies have specifically examined the neural basis of regret. For example, patients with lesions of the OFC, unlike normal controls, were not influenced by counterfactual outcomes in a simple gambling task, and did not report feeling regret when the outcome of a different choice would have been better (65). In a neuroimaging study, a task condition designed to increase the sense of agency for a decision resulted in decreased striatal activity after a loss (70). Another study found decreased ventral striatal activity when the alternative choice in a gamble would have been superior (a regret-related outcome), compared to when the alternative choice would have been inferior (a relief-related outcome) (71). In a study which manipulated the degree of the subject’s responsibility for an outcome, the amygdala was more highly activated in response to regret-related outcomes when subjects had higher levels of objective and subjective responsibility (72). Another study found enhanced activity in the medial orbitofrontal region, the ACC, and the hippocampus with increasing regret (calculated as the actual outcome subtracted from the superior alternative outcome) (13). In the same study, amygdala and medial orbitofrontal activity increased during anticipation of regret. One study comparing regret with disappointment found that both emotions caused activation of the anterior insula and dorsomedial PFC, but regret specifically activated the OFC (31). In summary, neuroimaging studies have shown that areas known to be involved in decision-making (20, 21), including the OFC, amygdala, nucleus accumbens, and medial PFC, all respond not only to actual outcomes, but to actual outcomes in comparison to counterfactual outcomes. When the alternative outcome would have been better, reward areas such as the striatum show lower activation. Contrastingly, the amygdala, anterior cingulate, insula, and OFC appear to show increased activity in response to regret-related outcomes. Furthermore, a lesion study, along with a number of neuroimaging studies, suggests that the OFC may play a critical role in counterfactual-related regret (65, 67). Generally, the OFC is believed to encode the expected value of various choices in the context of the organism’s current goals (73, 74). The OFC is thought to assign a value to a stimulus in a manner which is modulated by the internal state of the organism (75). For example, the value of food depends on whether the organism is hungry. It is possible that the OFC plays a similar role in modulating the value of a stimulus depending on counterfactual outcomes (such as a decrease in valuation of an outcome when a better outcome could have been obtained). In general, the OFC plays a role in linking cognitive processes to emotion, and counterfactual thinking may be one cognitive process which modulates emotion in a top-down fashion via the OFC (13). Further research may help elucidate the relationship of counterfactual thinking with the overall function of the OFC.

### Fictive error

One line of research investigating neural processing of counterfactual outcomes draws from computational accounts of reinforcement learning, in which reward prediction errors drive learning. This line of research extends reinforcement learning to also include “fictive errors” in addition to prediction errors, which are both thought to drive learning. The term fictive error refers to the difference between an actually experienced outcome and a counterfactual outcome (what would have happened if a different choice had been made). It is therefore directly related to regret and relief. A number of studies have shown that fictive error signals both drive subsequent behavior and correlate with neural activity in specific brain regions. For example, one fMRI study used a sequential investment task in which the subject experienced an outcome for each decision, but was also aware of the counterfactual outcome if the choice had been different (76). The ongoing difference between the best possible counterfactual outcome and the actually experienced outcome (the fictive error signal) influenced subsequent choices in the task – larger fictive errors led to larger changes in investment behavior. Additionally, the fictive error signal correlated with activity in the ventral caudate, a reward-related region that receives dopaminergic input. A study employing similar methods to examine differences between smokers and non-smokers found that both smokers and non-smokers showed a robust fictive error signal in the caudate (77). However, smokers, unlike non-smokers, were not guided by this fictive error signal in terms of behavior. Fictive reward signals have also been investigated in animals; a study using monkeys found that neurons in the ACC respond both to actually experienced rewards and to fictive rewards (counterfactual outcomes of choices that were not made) (78).

## Self-Esteem

Self-related processing is an important influence on how counterfactual thinking leads to a variety of emotions. In particular, exaggerated counterfactual-based emotions can lead to profoundly painful experiences such as shame and guilt (26). Social scientists have long been interested in the concept of self-esteem. Research has suggested that self-esteem may play an important role in human emotion, social relationships, and mental illness. What follows is a selective review of the large literature on self-esteem, focusing on the distinction between level of self-esteem and *security* of self-esteem, the relationship between self-esteem and counterfactual thinking, and neuroimaging studies related to self-esteem and self-related processing more generally.

While a great deal of attention has been focused on the distinction between high and low self-reported self-esteem, the relevance of this distinction in regard to psychological functioning has been called into question (79–81). An alternative to *level* of self-esteem is the *security* of self-esteem. A body of literature indicates that *secure* high self-esteem, rather than high self-esteem in itself, is associated with positive psychological functioning. Conversely, *fragile* self-esteem is related to poor functioning in a number of domains (79). There are two important attributes of fragile self-esteem: it is unstable, and it is contingent on external factors. Unstable or labile self-esteem tends to fluctuate from day to day or within each day. Contingent self-esteem remains high only if individuals meet internal or external standards of worthiness; for example, it is only high in the event of an achievement or compliment (79, 82). Contingent and labile self-esteem are related in that people with self-esteem which is contingent on external factors also tend to have more unstable self-esteem (83–86). People with fragile high self-esteem are more prone to self-glorification, aggressiveness, and verbal defensiveness (a tendency to distort self-related information) (79, 87–89). Additionally, these individuals have lower overall levels of psychological well-being; comparatively, individuals with secure high self-esteem are more autonomous, have a clearer sense of meaning in their lives, relate more effectively with their physical and social environments, and are more self-accepting (79). This evidence for the negative consequences of fragile self-esteem suggests that individuals with this type of brittle self-representation are more sensitive to the affective consequences of self-related information and outcomes. Their actual and anticipated affective reactions to self-related information appears to result in several maladaptive behavioral patterns, such as increased aggressiveness, as well as overall poorer functioning.

### Self-esteem, counterfactual thinking, and regret

Self-esteem has been related to counterfactual thinking. One study examined counterfactuals which were spontaneously generated by individuals who were high and low in self-esteem after imagining themselves interacting with another person (90). After failure, subjects low in self-esteem were more likely to imagine themselves behaving differently (i.e., imagine how they could have acted differently to produce a better outcome). While the authors do not use the term “regret,” their results suggest that individuals with low self-esteem were more likely to feel regret by focusing on a superior counterfactual outcome. Another study examined the interaction between self-esteem and mood in counterfactual thinking (91). When in negative moods, subjects with high self-esteem imagined counterfactuals which were worse than the actual outcome (which would tend to result in relief); subjects with low self-esteem imagined counterfactuals which were better than the actual outcomes (which would tend to result in regret or disappointment). Together, these studies suggest that people with low self-esteem compared to individuals with high self-esteem are more likely to focus on how things could have gone better.

The effect of self-esteem and regret on decision-making has been specifically examined. A study comparing low and high self-esteem subjects found that differences in decision-making only emerged when subjects expected to receive feedback on the outcomes of their decisions (92). When feedback was expected, subjects with low self-esteem were more likely to make choices which avoided the possibility of large regrets. For example, they were more likely to choose a certain $8 rather than a 66% chance of $12 to avoid the regret associated with losing all the money. When not expecting to receive feedback on the outcomes, the difference between high and low self-esteem subjects disappeared, indicating the difference was not due merely to risk-aversion. This study suggests that a motive to protect self-esteem from the threat of regret can influence decision-making. Further research is needed examining the impact of secure vs. fragile self-esteem, rather than merely level of self-esteem, on counterfactual thinking and regret.

### Self-esteem and the brain

Neuroimaging has been used to investigate self-related processing in general and self-esteem in particular. Self-related processing compared to processing not related to the self appears to activate midline brain structures including medial prefrontal and posterior cingulate cortices (93–95). Several studies have investigated the neural response to social feedback in people with low self-esteem. In these individuals, social rejection is associated with greater activation in the dorsal anterior cingulate cortex (dorsal ACC) (96). Subjects with low self-esteem had enhanced activity in ventral anterior cingulate cortex (vACC)/mPFC in response to positive feedback (97). Another study showed that subjects with lower state self-esteem in response to social feedback showed greater activity in the dorsal ACC and anterior insula (98). Subjects whose self-esteem decreased from prescan to postscan vs. those whose self-esteem did not decrease showed greater medial prefrontal cortical activity. Overall, it appears that state and trait level of self-esteem modulates the impact of events on the activation of decision-making areas. Further research is needed examining the relationship of security vs. fragility of self-esteem with neural activation, rather than absolute level of self-esteem alone.

There are specific subpopulations of individuals with markedly unstable and contingent self-esteem, prominently including borderline personality disorder and narcissistic personality disorder, and the brain bases of these disorders are beginning to be investigated. For example, borderline personality disorder is characterized by markedly unstable self concept including unstable self-esteem (99). Studies have indicated that patients with borderline personality disorder may have abnormalities in the regions noted above. For example, a volumetric MRI study found that patients with borderline personality disorder had lower volumes in the amygdala, left OFC, and right ACC, along with the hippocampus (100). Functional studies have also found decreased ventromedial PFC activity including medial OFC and subgenual ACC during behavioral inhibition in the context of negative emotion (101) and reduced activation of the subgenual and dorsal ACC and the amygdala and greater activation in the insula and posterior cingulate during negative emotionality (102). Patients with narcissistic personality disorder tend to exhibit contingent and defensive self-esteem (103). An fMRI study found that high narcissistic subjects showed less deactivation of the insula during empathy (104), and a structural MRI study found lower volume in left anterior insula in patients with narcissistic personality disorder (105). The relationship between unstable, contingent self-esteem, and neural processes in patients with borderline personality disorder and narcissistic personality disorder remains to be established.

## Decision-Making, Counterfactual Thinking, and Self-Esteem in Depression

### Depression and counterfactual thinking

Some research has examined the relationship of depression with altered counterfactual thinking. One study examined the types of counterfactual thoughts generated by subjects with mild depression. When mildly depressed subjects considered how things could have gone better, they tended to focus on factors that were more controllable than did non-depressed subjects (106). This is interpreted as reflecting an attempt on the part of mildly depressed individuals to increase their sense of control, a tendency that has been shown in prior studies (107, 108). Presumably, however, focusing on controllable factors after a negative outcome results in increased regret by highlighting how one could have prevented the outcome. Contrasting with this study, a later study found that *severely* depressed subjects tended to generate counterfactuals that were less controllable, less reasonable, and more characterological in nature (109). These counterfactual thoughts are argued to be similar to other types of negative cognitive distortions in severe depression.

### Depression and regret

Several studies have examined the relationship between depression and regret. Scores on a regret-proneness scale are positively correlated with the Beck Depression Inventory (14). In a sample of older adults, the intensities of loss-and-grief related regrets were correlated with scores on the Geriatric Depression Scale, although regret only explained a small amount of the variance in depression scores (110). Mildly depressed subjects making a hypothetical hiring decision exhibited more regret after a decision, regardless of which decision they actually made (111). This study assessed depression by having a random sample of college undergraduates fill out the Beck Depression Inventory; no psychiatric interview was performed. Another study, using a telephone survey, found that regret was associated with anhedonic depression and anxious arousal (112). Anhedonic depression was assessed with an eight-item scale rather than a full psychiatric interview. Contrasting with these results, a study using a computerized decision-making task found that individuals with major depression experienced less regret, an effect that was particularly related to self-reported apathy scores (113). This study used DSM-IV criteria to identify depressed subjects. Given the limited and inconsistent evidence about the relationship between depression and regret, further research is clearly needed to investigate this issue.

### Depression and self-esteem

Depression is closely related to self-esteem. The role of feelings toward the self has been an important consideration within diverse theoretical perspectives, including psychoanalytic and cognitive theories of depression (114–118). The DSM-V includes feelings of worthlessness as a symptom of major depression (119). Low levels of self-esteem have been related to prediction of depressive episodes as well as course and recovery (120, 121). However, beyond average level of self-esteem, *instability* of self-esteem is a crucial factor in depression (118, 122). In particular, research has consistently found that unstable self-esteem is a better prospective predictor of depression than low trait self-esteem (15, 16, 122–125). Unstable self-esteem also interacts with stressful life events in predicting depression; those who have unstable self-esteem are more likely to respond to a stressful event by becoming depressed (15, 16, 122, 126).

### Alterations in decision-related brain areas in depression

The literature on the neurobiology of depression is vast, and a summary of findings in this area is outside the scope of this review. However, it is useful to address evidence for abnormalities in decision-related brain areas in depression. Because these same brain areas also play a role in counterfactual thinking, this research can inform our model of abnormal counterfactual processing in decision-making in depression. It is important to note the heterogeneous nature of research on depression, given differences in study populations in terms of severity, chronicity, inpatient vs. outpatient samples, concomitant treatments, comorbid disorders, and other differences. There have been hundreds of fMRI studies demonstrating abnormalities in decision-related brain areas in depression, many of which use decision-making and reward task. For a more comprehensive discussion of this area, there are several recent reviews (127, 128).

One set of areas which have been shown to exhibit altered activity in depression are those that have also been implicated in encoding subjective value in decision-making tasks including the ventral striatum and OFC (20). In a neuroimaging study, individuals with depression showed decreased activity in regions of the ventral striatum in response to positive stimuli (129). Another study found a lack of activity in the medial caudate and ventromedial OFC in response to feedback on cognitive tasks (130). In a study of pediatric MDD, children with depression showed less activation of the caudate and regions of the OFC in a decision-making task (131). Depressed individuals show functional abnormalities in posterior lateral and medial OFC (132). In contrast, activity in anterolateral OFC and ventromedial frontal polar cortex is increased in depression (132). It is important to note the evidence reviewed above that, in normal subjects, activity in these same brain areas is modulated not only by a stimulus or outcome itself, but by unrealized alternatives.

Depression has also been associated with functional abnormalities of the amygdala, an area which plays a key role in stimulus processing, attention, and emotional learning (127, 133, 134). While research examining a specific role for the amygdala in altered decision-making in depression is limited, a number of studies have demonstrated amygdala dysfunction in tasks involving response to emotional stimuli and emotion regulation. For example, patients with depression show greater amygdala activity when anticipating negative emotional stimuli (135). A study of young subjects with depression (ages 15–24 years) found increased activation of the amygdala in response to positive social feedback (136). Depressed adolescents also have increased amygdala activity during an emotion regulation task (requiring them to notice and maintain an emotional reaction) (137). Multiple neuroimaging studies have found that depressed patients have increased amygdala activity in response to sad faces, thought to reflect a processing bias in depression (127, 138, 139). As discussed above, the amygdala has been implicated in counterfactual thinking and regret (13, 68, 72). Further research is needed to explore a possible role of altered amygdala function in decision-making, counterfactual thinking, and regret in depression.

Depression is also associated with altered functioning of the PFC. Some research has focused on alterations in this region during decision-making tasks, although this is still somewhat limited. Depressed subjects had decreased activation of the middle frontal gyrus during reward selection and anticipation in a gambling task (140). A decision-making study found that healthy adolescents, but not depressed adolescents, showed a negative correlation between activity in prefrontal areas and high-risk behavior (141). This suggests alteration of prefrontal function in risk and reward in depression, although further research is needed to clarify the implications of this finding. Overall, depression appears to be associated with reduced activity in the dorsolateral PFC, which is implicated in cognitive control; this reduced activity is specifically associated with psychomotor retardation and anhedonia (127, 128). Contrastingly, depressed subjects show increased activity in the ventromedial PFC, which is involved in generation of emotions, autonomic regulation, pain, and social behavior, among other functions (127, 128).

Finally, depressed individuals have alterations in functioning of the ACC. One study found decreased activity of rostral cingulate gyrus during reward selection and anticipation in a gambling task (140). An fMRI study of pediatric depression using a gambling task found decreased ACC activity during the decision phase, especially during a high-magnitude reward condition (142). The same study also found decreased ACC activity during the outcome phase in response to losses and small gains. Contrastingly, a study of depressed adolescents found increased activity in the right caudal ACC during the selection phase of a monetary decision task (141). Other research has investigated ACC differences not specifically related to decision-making. For example, depressed subjects showed increased activation of subgenual ACC to emotional stimuli in an fMRI study (143). Dorsal ACC, however, appears to be less active in depression (144). Given the heterogeneity of findings, more research is needed to clarify the role of ACC in depression, which may differ by sub-region, task, and population.

## A Simple Model of the Role of Self-Esteem and Counterfactual Thinking in Depression

**Figure 1:**
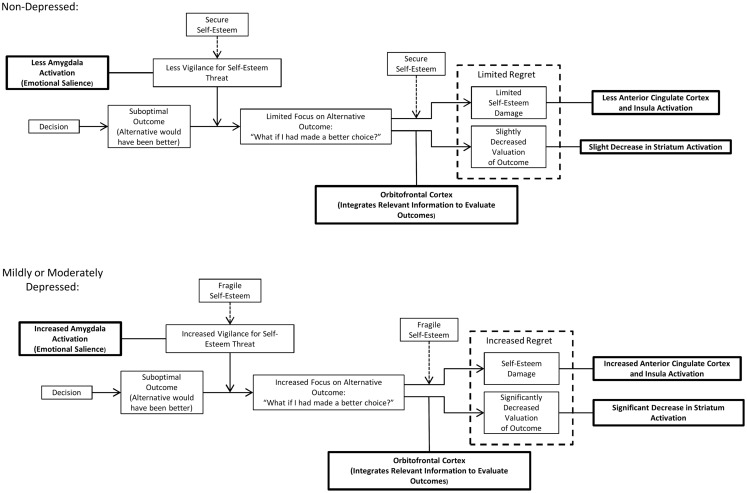
**A model of the role of self-esteem fragility and counterfactual thinking in mild to moderate depression**. In both non-depressed and mildly to moderately depressed individuals, learning that a different choice would have had a better outcome will result in self-related counterfactual thoughts, i.e., “what if I had chosen differently.” These thoughts will lead to feelings of regret, an aversive emotion. However, individuals with mild to moderate depression are more prone to regret because they have fragile self-esteem. In the model, fragile self-esteem will affect regret in two ways: (1) fragile self-esteem will result in greater vigilance for threats to self-esteem, including suboptimal decision outcomes. This vigilance is mediated by the amygdala, and increases attention focused on superior outcomes of alternate choices. This increased focus on the superior outcome will result in a decrease in subjective valuation of the actual outcome. The decrease in subjective value is mediated by the orbitofrontal cortex and is manifested by decreased striatum activation and (2) fragile self-esteem will be more highly damaged in response to learning that a decision outcome is suboptimal (a separate process from the increased vigilance for self-esteem threat in individuals with fragile self-esteem). This damage to self-esteem is affectively aversive and is mediated by increased activation of the anterior cingulate cortex and insula.

Based on the evidence reviewed above, a model is proposed here in which fragile self-esteem and counterfactual thinking play important and interrelated roles in decision-making in mild and moderate depression. The model (see Figure 1) applies to situations in which an individual has made a decision resulting in a suboptimal outcome; that is, an alternate choice would have produced a better outcome. In both non-depressed and depressed individuals, such a situation will result in self-related counterfactual thoughts, i.e., “what if I had chosen differently.” These thoughts will lead to feelings of regret, an aversive emotion. Regret is a normal emotion that is experienced at times by both depressed and non-depressed individuals. However, it is proposed here that regret is intensified in mild to moderate depression, and that an important contributor to this process is fragile self-esteem, which is found in mild to moderate depression. Within the model, fragile self-esteem affects regret through two separate processes.

The first process by which fragile self-esteem influences regret is through increased vigilance for potential threats to self-esteem. This process may be mediated by the amygdala, which is believed to be involved in the attribution of emotional salience and in emotional attention (133, 134). Importantly, increased amygdala activation is tied to depression (138) and regret (72). Learning that the outcome of one’s decision was suboptimal may result in self-blame and be threatening to self-esteem. The increased vigilance for threats to self-esteem in mild to moderate depression will therefore cause increased attention to be devoted to counterfactual scenarios in which an alternative choice would have produced a better outcome. This increase in attention to the superior counterfactual outcome then results in decreased subjective value of the actual outcome, in comparison to the counterfactual outcome. This process may be mediated by the OFC, which integrates information to evaluate outcomes and is involved in regret (31, 65, 73, 74). The result will be decreased activation of the striatum, reflecting a lower value attached to the actual outcome. This decrease in subjective valuation of the outcome is one of the components of the affective experience of regret as formulated in the model.

The second process by which self-esteem fragility influences regret is through the increased damage to self-esteem incurred when a different choice would have had a better outcome. Thoughts about a superior counterfactual outcome of a different choice will have greater effect on self-esteem when self-esteem is fragile. This will result in an aversive emotional experience. This process may be mediated by the ACC and insula, which have been shown to have higher activity in people with reductions in state self-esteem in response to social feedback (98). These regions have been associated with regret (13, 31) and have shown differential activation in depression (145). Within the model’s formulation of regret, the aversive damage to self-esteem is one of the components of the emotional experience of regret, along with the reduced value associated with the actual outcome of the decision.

Some aspects of the model are supported by current evidence, whereas others are more speculative. The model proposes two components of regret that affect people with depression: reduced subjective value of actual outcomes in comparison to superior alternatives, and damage to self-esteem that is mediated by self-esteem fragility. Regarding the first component, there are a number of studies on normal subjects, reviewed above, showing that the subjective value of an outcome is reduced when it is compared to superior hypothetical alternatives. Whether and how this differs in depression, however, is largely untested. For the second component, there is a sizable amount of evidence that fragile self-esteem is linked to a higher risk for depression. However, the connection between fragile self-esteem and regret has been almost entirely unexplored.

This model focuses primarily on individuals with mild to moderate depression. In severely depressed individuals, other process may contribute to dysfunction of decision-making, including severe cognitive distortions, apathy, and affective blunting (109, 113). It is even possible that severe apathy and blunted responsiveness to positive outcomes may *decrease* regret, due to reduced appreciation for a missed, positive outcome. This may help explain the discrepancy in studies investigating regret in depression, in which milder depression tends to be associated with increased regret, while at least one study finds that more severe depression is associated with decreased regret (although this needs more study) (14, 110–113). However, the model may be relevant to the development of severe depression, which may have been preceded by a period of mild to moderate depression or by a period in which the person was not depressed, yet possessed an underlying vulnerability to the development of depression in the form of fragile self-esteem.

The model generates a number of testable predictions. First, mildly and moderately depressed subjects should experience increased regret after learning an alternative decision would have produced a better outcome (e.g., as measured by self-report in a simple gambling task). The same finding would be expected for individuals with fragile self-esteem. In addition to self-report differences, depressed subjects may also show differences in the behavioral influence of “fictive error” signals, reflecting the difference between counterfactual and actual outcomes, in a task similar to the sequential investment tasks from previous fictive error studies (76, 77). Another expected finding is that mildly and moderately depressed subjects and those with fragile self-esteem will exhibit increased attention to superior counterfactual outcomes of alternative choices, when these are revealed by feedback. This might be measured by eye movement tracking, which has previously been used to measure attentional biases in mental illness including anxiety disorders and depression (146–148).

In terms of neuroimaging, it is predicted that individuals with mild to moderate depression and those with fragile self-esteem will show altered neural responses to learning that the outcome of a different choice would have been superior to the one which was actually received. Specifically, activity in OFC, ACC, the insula, and the amygdala will be increased, whereas activity in the striatum will be decreased. Several of these areas may also be active while making a choice and while anticipating the outcome; these may include the amygdala and OFC. It is also predicted that depressed individuals will show alterations in neural “fictive error” signals, which may be detected in fMRI studies (76, 77).

This model is not intended to be comprehensive, and certainly leaves out many important factors in the development of depression. However, it presents a framework for the relationship between several processes which are thought to play important roles in depression. Furthermore, it generates testable hypotheses which might help to further clarify the processes involved in this disorder. Finally, it may assist in the development of interventions specifically targeting the altered processing of counterfactuals in depression.

The model relates to decision-making, a growing field with contributions from economists, psychologists, and neuroscientists. The neural underpinnings of decision-making processes are beginning to be illuminated, and these findings may be useful to understanding the neurobiology of depression. It is interesting to note that the brain areas involved in decision-making also appear to play a role in counterfactual thinking, self-esteem, and depression. Further elucidation of the neural correlates of these processes, and their interrelationships, could be a step toward a more integrated theory of depression.

## Clinical Implications

This review has presented previous research on counterfactual thinking, depression, and self-esteem, and presented a model of how counterfactual thinking and self-esteem abnormalities may be linked in depression. The aim is to establish a better mechanistic understanding of the specific processing dysfunctions in this disorder, which will help to develop better treatments that are targeted specifically toward correcting these dysfunctional processes. Specifically, treatment may attempt to diminish the influence of counterfactual comparisons on self-representation, thereby decreasing levels of regret. There are several possible avenues for treatment approaches. These may include exposure protocols to reduce the affective impact of regretted outcomes, cognitive approaches to challenge unrealistic counterfactual scenarios, or training to reduce attentional bias toward superior alternative outcomes. One possible treatment method to accomplish these goals would be a computerized training approach. This might involve a computerized decision-making game to facilitate exposure to regretted outcomes or to help correct attentional biases. Such interventions are examples of treatment approaches which are targeted toward specific pathologic processes identified in research (149). This is somewhat similar to a previous group-based approach targeting memory dysfunction (150) and a computerized approach targeting negative cognitive bias (151). Given that the dysfunctional counterfactual and self-esteem processes may be linked to specific brain regions, it may even be possible to target these dysfunctions with biological approaches including repetitive transcranial magnetic stimulation (rTMS), transcranial direct-current stimulation (tDCS), and deep brain stimulation (DBS) targeting the highlighted regions. Overall, it is hoped that adding to our knowledge of the specific processes involved in depression will help identify and treat the particular difficulties faced by those with this common and burdensome disorder.

## Conflict of Interest Statement

The authors declare that the research was conducted in the absence of any commercial or financial relationships that could be construed as a potential conflict of interest.
